# Adapting contingency management to link and retain HIV-infected transgender women of color in HIV care

**DOI:** 10.1186/1940-0640-10-S1-A53

**Published:** 2015-02-20

**Authors:** Cathy J Reback, Jesse B Fletcher, Kimberly A Kisler

**Affiliations:** 1 Friends Research Institute, Los Angeles, CA, 90028, USA

## Background

HIV prevalence among transgender women is estimated to be 50 times greater than that of nontransgender adults, and yet HIV-positive transgender women exhibit low rates of linkage to and retention in HIV primary care, trends which are exacerbated among substance-using and minority transgender women. Transgender women experience a number of psychosocial challenges specific to their gender identification, including discrimination, prejudice, stigmatization, and social/economic marginalization. Such issues stand as obstacles to engagement and retention in medical care, substance abuse treatment, and mental health and social services. Transgender women report discrimination and/or blatant verbal abuse at standard health-care facilities. In response to this health disparity, *The Alexis Project* combines an innovative application of Contingency Management (CM), in conjunction with Peer Health Navigation (PHN), to improve linkage to and retention in HIV primary care, and achieve viral load suppression among HIV-infected transgender women of color in Los Angeles County.

## Methods

Between February 2014 and February 2016, the study will enroll 140 HIV-infected transgender women of color who have either: 1) never been in HIV care; 2) have dropped out of HIV care; or 3) are nonadherent to current HIV medications. Data collection began in February 2014; current enrollment is N = 34. During the course of the 18-month intervention, participants can earn CM rewards for confirmed linkage to HIV primary care (i.e., first HIV care appointment), retention in HIV care (i.e., quarterly HIV care appointments), and reaching HIV milestones (i.e., fill antiretroviral [ART] medication prescription, medication adherence as confirmed by log reductions; HIV viral load suppression). If a participant attends all of her HIV care visits and reaches each verified HIV milestone, she can earn $500 in CM rewards over the 18-month intervention.

## Results

To date, most participants self-identified as African American/black (47.1%), Hispanic/Latino (32.4%), or multi/other (17.6%), and the mean participant age was 35.4 (SD = 7.6). Less than half of the participants graduated from high school (44.1%), half reported current homelessness (50%), and slightly over half reported a prior AIDS diagnosis (52.4%). Most reported using Medicare/Medicaid/other public insurance (79.4.1%), and/or attending a community or county clinic to receive health care (50%), and just over one-third reported receiving HIV primary care in the previous 6 months (38.1%). In the past 6 months, 47.1 percent reported using methamphetamine, 27.3 percent reported ever using an amphetamine, 25 percent reported using cocaine, and lifetime injection drug use was 17.7 percent. Slightly under half (42.9%) reported having ever been on ART. To date, 66.7 percent of the participants have been linked into HIV care (i.e., attended their first HIV medical appointment); 41.2 percent have received their initial laboratory results and filled their ART prescription. The mean enrollment-to-linkage time has been 22.4 days (SD = 31.4; range = 0–148 days).

## Conclusions

Preliminary data indicate that CM, in combination with PHN, will be effective in linking HIV-infected transgender women of color into HIV primary care. Longitudinal data of retention in care and ART medication adherence will provide further indication of whether this novel application of CM can produce viral load suppression and improve the health outcomes of HIV-infected transgender women of color.

